# Isolated Infraspinatus Myositis after Intramuscular Vaccine Administration

**DOI:** 10.1155/2022/1363462

**Published:** 2022-08-05

**Authors:** Eric R. Samuelson, Joseph M. Bano, Heath P. Gould, Richard G. Levine

**Affiliations:** ^1^Georgetown University School of Medicine, Washington, District of Columbia, USA; ^2^Department of Orthopaedic Surgery, MedStar Union Memorial Hospital, Baltimore, Maryland, USA

## Abstract

**Case:**

A 74-year-old female developed left shoulder pain after receiving an influenza vaccine. Her initial physical exam was suggestive of subacromial bursitis, and a corticosteroid injection into the subacromial space resulted in a 50% improvement in her pain. Subsequent MRI demonstrated myositis isolated to the infraspinatus muscle. She was successfully treated with anti-inflammatory medication and physical therapy.

**Conclusion:**

Shoulder injury related to vaccine administration (SIRVA) is a rare clinical complication, and myositis in the rotator cuff musculature has not been previously reported. Proper administration of intramuscular vaccinations should be emphasized to prevent injury to structures surrounding the shoulder joint.

## 1. Introduction

Shoulder injury related to vaccine administration (SIRVA) is associated with improper vaccine administration technique and can manifest as trauma or inflammation to the bursae, tendons, or ligaments of the shoulder joint 1, 2. Injury to the radial and axillary nerves have also been reported after intramuscular vaccine administration 2. These complications appear to be influenced by needle length, location of injection site, and patient characteristics such as body mass index (BMI) 1–3. We present a case of a patient developing an isolated myositis of the infraspinatus muscle after administration of an influenza vaccine.

## 2. Case Report

A 74-year-old Caucasian female with no significant past medical history presented to the orthopaedic sports medicine clinic for evaluation of left shoulder pain. The patient reported receiving an influenza vaccine 14 days before presentation and said she noticed the onset of shoulder pain shortly after receiving the vaccine. Her presenting symptoms included pain in the subdeltoid region and difficulty lifting her arm above her head and behind her back. She could not recall any other trauma or inciting events that might have played a role in the development of these symptoms. She reported no antecedent shoulder pain or surgeries in the affected shoulder before presentation. At presentation, her symptoms had persisted for 2 weeks despite the use of nonsteroidal anti-inflammatory drugs (NSAIDS).

Physical examination revealed a healthy-appearing woman in no acute distress. The skin was intact with no lesions. Her cervical spine was normal with full range of motion. There were no signs of cervical radiculopathy. Her left shoulder examination revealed full passive and active range of motion and pain with active abduction, forward flexion, and external rotation. The shoulder was nontender to palpation. She reported significant discomfort with Neer and Hawkins impingement maneuvers. Her sensory and motor function was intact throughout the affected upper extremity, and she had a palpable radial pulse.

Plain radiographs of the left shoulder obtained at the initial clinic visit demonstrated no evidence of fracture, degenerative joint disease, or other bony lesions. The patient's presentation was consistent with subacromial bursitis. A corticosteroid injection of 1 cc Kenalog (40 mg) and 9 cc 1% lidocaine without epinephrine was administered to the subacromial space for diagnostic and therapeutic purposes in the usual sterile fashion. NSAIDs were recommended for further pain control, and she was given a prescription for physical therapy.

One month later, the patient continued to experience symptoms and therefore returned to the clinic for a follow-up visit. She reported a 50% improvement in her pain after the injection.

Physical examination showed a full range of motion in her left shoulder, though she still experienced pain with the provocative rotator cuff and impingement maneuvers. Due to her persistent symptoms after corticosteroid injection, she was advised to undergo noncontrast MRI of the shoulder to evaluate the integrity of her rotator cuff.

**Figure 1:**
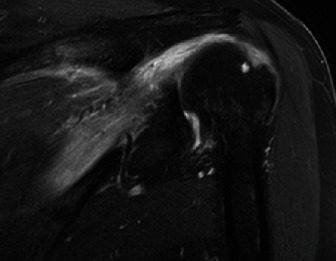
Coronal T2 MRI of the left shoulder demonstrating hyperintensity throughout the infraspinatus muscle, consistent with myositis.

**Figure 2:**
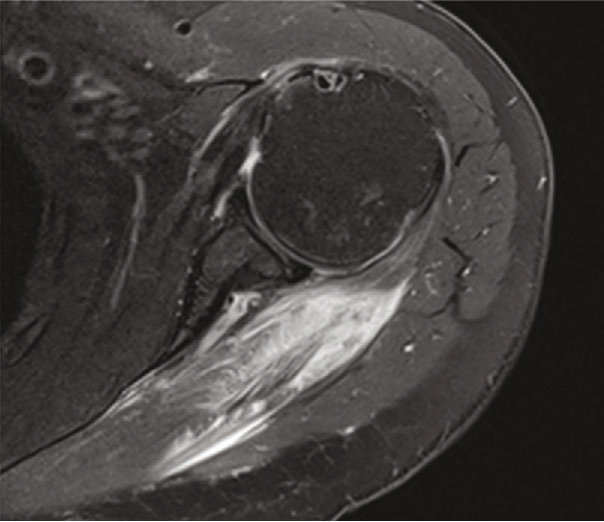
Axial T2 MRI of the left shoulder demonstrating hyperintensity throughout the infraspinatus muscle, consistent with myositis.

The patient returned to the clinic 1 week later to discuss the results of her MRI, which demonstrated marked edema throughout the infraspinatus muscle (Figures 1 and 2) and a 3 cm full-thickness supraspinatus tendon tear. There was also an effusion present in the glenohumeral joint. She was advised to continue physical therapy and return in 3 weeks for repeat examination and imaging.

Figures 1 and 2 show coronal and axial views, respectively, on T2 MRI with marked hyperintensity of the infraspinatus as well as a 3 cm full-thickness supraspinatus tendon tear. There is also an effusion present in the glenohumeral joint.

At follow-up, the patient had full range of motion of the left shoulder, though she had pain with external rotation. Repeat shoulder MRI without contrast revealed a substantial interval decrease in the infraspinatus edema. She was advised to continue with her physical therapy protocol and follow-up in 2 months.

The final follow-up visit was conducted via telehealth. The patient noted resolution of her shoulder pain and continued weakness with overhead activities. She had completed the recommended 6-week physical therapy program and was satisfied with her recovery. She was not interested in pursuing further management or surgical intervention for weakness. She was encouraged to return for further discussion if that symptom began to limit her daily activities.

## 3. Discussion

Although a definitive causal relationship was not established in this case, the temporal relationship between the vaccine administration and the development of the patient's shoulder symptoms suggests a vaccine-induced etiology and support the conclusion that isolated infraspinatus myositis was the primary source of her shoulder pain.

Generally, vaccine-related shoulder injuries are caused by administration into the proximal third of the deltoid muscle, use of a needle that is too large for the patient, or not inserting the needle at a 90-degree angle relative to the skin 1. Any of these factors can result in the inadvertent administration of the vaccine into the shoulder joint, leading to an exaggerated inflammatory response and/or trauma to the intra-articular structures. It has been speculated that preformed antibodies are present within the bursa and synovial tissue of the shoulder joint as a result of previous infection or vaccination. These antibodies can react with components of intramuscular vaccines, resulting in an immune response that has been reported to last 6 weeks in animal models 4. A prior study by Atanasoff et al. investigated the potential role of poor vaccine administration technique in the development of SIRVA by placing a needle into a patient's self-reported injection site during an elective shoulder arthroscopy after a presumed SIRVA.

Arthroscopic examination demonstrated inflamed and scarred bursa and thickened rotator cuff tendons in the replicated path of the original intramuscular vaccination 1. The complications that can occur as a result of SIRVA appear to be preventable by adhering to proper intramuscular vaccination technique 2.

The differential diagnosis in the context of SIRVA is broad. The patient's symptoms in this case could have conceivably been attributed to a rotator cuff tear, tendonitis, or bursitis. Her 50% symptomatic relief after a subacromial corticosteroid injection may support subacromial bursitis as a contributing factor to her symptoms. She also had an effusion of the glenohumeral joint, possibly due to the supraspinatus tear discovered on MRI. This tear could have been a previously asymptomatic rotator cuff tear that became symptomatic due to vaccine-induced synovial inflammation 1, 5. However, the patient's edema in the infraspinatus, without any corresponding history of trauma, suggests that this myositis was the predominant etiology of her symptoms. Although previous case studies have reported deltoid myositis after the administration of intramuscular vaccines 6, 7, the authors are not aware of any report of isolated infraspinatus myositis after vaccine administration. This finding suggests that many different structures surrounding the shoulder joint can be affected by improper vaccine administration, and SIRVA should be considered a differential diagnosis in patients presenting with prolonged shoulder pain following intramuscular vaccination.

Healthcare providers should be familiar with SIRVA and its potential complications because patients frequently present with shoulder pain after intramuscular vaccination 6. Most patients with SIRVA can be managed successfully with nonoperative measures such as NSAIDs, corticosteroid injections, and physical therapy 8. Still, the symptoms associated with SIRVA can last for months, and patients should be informed of this risk prior to receiving an intramuscular vaccination 8. From a public health perspective, it is important to provide adequate training to health professionals regarding the proper techniques for vaccine administration to decrease the incidence of SIRVA and prevent its associated complications.

Our case report is limited by lack of data regarding the length of the needle used, distance from the patient's skin to bursa, and patient-reported location of the vaccination administration site. Moreover, a biopsy of the infraspinatus muscle was not performed to confirm the presence of myositis.
